# Supplementary material for: Polyketide synthase gene clusters in the ericoid mycorrhizal fungus Oidiodendron maius: genome-wide prediction, expression profiling, and functional characterization of knockout mutants
Source: Front Plant Sci. 2026 Sep 16;17:1924430. doi: 10.3389/fpls.2026.1924430 (PMC13623903; doi:10.3389/fpls.2026.1924430)
Supplement: Supplementary file 1 [file SupplementaryFile1.zip › Supplementary Materials/Supplementary Folder 1.zip!/Oidma1_AssemblyScaffolds_Repeatmasked/knownclusterblast/cluster12/jgi.p_Oidma1_157872_mibig_hits.html]

| MIBiG Protein | Description | MIBiG Cluster | MiBiG Product | % ID | % Coverage | BLAST Score | E-value |
| --- | --- | --- | --- | --- | --- | --- | --- |
| ACD39772.1 | major\_facilitator\_superfamily\_transporter | BGC0000134 | Radicicol\_biosynthetic\_gene\_cluster | 45 | 98.4848484848 | 404 | 1.2e-112 |
| KFH48645.1 | putative\_transporter\_-like\_protein | BGC0000317 | Cephalosporin\_biosynthetic\_gene\_cluster | 44 | 98.4848484848 | 396 | 3.2e-110 |
| BAK26557.1 | putative\_MFS\_multidrug\_transporter | BGC0000977 | Cyclopiazonic\_acid\_biosynthetic\_gene\_cluster | 45 | 99.1341991342 | 385 | 9.7e-107 |
| AGO86666.1 | putative\_MFS\_transporter | BGC0001255 | Equisetin\_biosynthetic\_gene\_cluster | 41 | 99.3506493506 | 335 | 1.2e-91 |
| AGO65989.1 | putative\_MFS\_transporter | BGC0000992 | Fusaridione\_A\_biosynthetic\_gene\_cluster | 37 | 100.649350649 | 308 | 1.2e-83 |
| ADI24938.1 | VrtL | BGC0000168 | Viridicatumtoxin\_biosynthetic\_gene\_cluster | 35 | 94.8051948052 | 266 | 6.6e-71 |
| CAP95407.1 |  | BGC0001404 | Sorbicillin\_biosynthetic\_gene\_cluster | 35 | 98.0519480519 | 264 | 1.9e-70 |
| ALI92656.1 | MRR1\_Major\_Facilitator\_Superfamily\_(MFS)\_protein | BGC0001338 | Citrinin\_biosynthetic\_gene\_cluster | 35 | 99.5670995671 | 261 | 2.1e-69 |
| KFH48706.1 | putative\_MFS-type\_transporter-like\_protein | BGC0000317 | Cephalosporin\_biosynthetic\_gene\_cluster | 36 | 96.7532467532 | 251 | 2.2e-66 |
| EAU32817.1 | conserved\_hypothetical\_protein | BGC0000160 | Terreic\_acid\_biosynthetic\_gene\_cluster | 36 | 89.1774891775 | 228 | 2e-59 |
| CCH26293.1 | putative\_MFS-type\_transporter | BGC0001158\_c4 | Bikaverin\_biosynthetic\_gene\_cluster | 32 | 101.298701299 | 219 | 7.1e-57 |
| ABK64181.1 | MFS\_transporter | BGC0001247\_c4 | Cercosporin\_biosynthetic\_gene\_cluster | 33 | 98.7012987013 | 218 | 1.6e-56 |
| CCT67996.1 | bikaverin\_cluster-efflux\_pump | BGC0000030 | Bikaverin\_biosynthetic\_gene\_cluster | 32 | 101.298701299 | 216 | 7.8e-56 |
| ABS57485.1 | putative\_MFS\_multidrug\_transporter | BGC0000048 | Dothistromin\_biosynthetic\_gene\_cluster | 29 | 100.649350649 | 155 | 2.1e-37 |
| XP\_011392678.1 | hypothetical\_protein | BGC0001281 | Ustilagic\_acid\_biosynthetic\_gene\_cluster | 29 | 75.7575757576 | 151 | 2.4e-36 |
| AMY15073.1 | MFS\_transporter | BGC0001339 | Squalestatin\_S1\_biosynthetic\_gene\_cluster | 23 | 97.4025974026 | 84 | 4.6e-16 |
| CAP12594.1 | elloramycin\_permease | BGC0000219 | Elloramycin\_biosynthetic\_gene\_cluster | 27 | 36.3636363636 | 70 | 6.9e-12 |
